# Depressive Syndrome as a Mediator Between Chronic Pain and Alzheimer's Disease in Latin American Populations

**DOI:** 10.1002/alz70857_105735

**Published:** 2025-12-25

**Authors:** Nickole P. Marin‐Díaz, Joaquín Migeot, Maria‐Eugenia Rippes, Hernan Hernandez, Ariel Caviedes, Jessica L Hazelton, Lorena Oyanedel, Teresita Ramos Franco, Loreto Olavarria, Agustin Ibanez, Nilton Custodio, Rosa Montesinos, Martin Alejandro Bruno, José Alberto Avila Funes, Andrea Slachevsky, Claudia Duran‐Aniotz, Carolina Gonzalez‐Silva

**Affiliations:** ^1^ Latin American Brain Health Institute (BrainLat), Universidad Adolfo Ibañez, Santiago, Chile; ^2^ Pontificia Universidad Católica de Chile, Santiago, Chile; ^3^ Hospital del Salvador, Santiago, Metropolitana, Chile; ^4^ Escuela de Psicología, Universidad Mayor, Santiago, Chile; ^5^ Global Brain Health Institute (GBHI), University of California San Francisco (UCSF); & Trinity College Dublin, Dublin, Ireland; ^6^ Unidad de Investigación, Instituto Peruano de Neurociencias, Lima, Lima, Peru; ^7^ Unidad de Investigación, Instituto Peruano de Neurociencias, Lima, Peru; ^8^ Instituto de Ciencias Biomédicas (ICBM) Facultad de Ciencias Médicas, Universidad Catoóica de Cuyo, San Juan, San Juan, Argentina; ^9^ Instituto Nacional de Ciencias Médicas y Nutrición Salvador Zubirán, México, DF, Mexico; ^10^ Memory and Neuropsychiatric Clinic (CMYN), Neurology Service, Hospital del Salvador and Faculty of Medicine, Universidad de Chile, Santiago, Chile

## Abstract

**Background:**

Chronic pain (CP) and depression frequently co‐occur in the elderly, severely impacting individuals with Alzheimer's disease (AD). Their overlapping neurobiological pathways exacerbate each other, accelerating functional decline and worsening cognitive deficits. Untreated CP and depression may even contribute to dementia progression, while adverse social determinants of health (SDH) further compound their effects. However, the triadic relationship between CP, depression, and AD remains poorly understood, particularly in Latin American (LA) populations, where health disparities and environmental factors may shape outcomes. This study explores their interplay in this underrepresented context.

**Method:**

A cohort of 320 older adults, including 146 individuals with AD and 174 healthy controls (HC), was recruited from Chile, Argentina, Mexico, and Peru. The sample was sex‐balanced, and all participants provided informed consent in accordance with the Declaration of Helsinki. Recruitment was facilitated through the Multicenter Consortium to Expand Dementia Research in Latin America (ReDLat), utilizing standardized protocols across all sites. Data collection encompassed neuropsychological assessments, CP and depression evaluations, SDH measures, and demographic factors. CP was assessed through indicators such as pain frequency, intensity, and symptom severity, while depression was evaluated using validated scales.

**Result:**

Structural equation modeling (SEM) revealed that depression significantly mediated the relationship between CP and AD. A marginally significant positive association was identified between depression and CP (*p* = 0.050), and a strong association was observed between depression and AD (*p* = 0.001). CP, as represented by pain frequency, intensity, and symptom severity, demonstrated high explanatory power (*R* ^2^ = 97.6%). Depression also showed a strong association with dementia risk (*R* ^2^ = 96.4%). Furthermore, unfavorable SDH were positively correlated with higher CP and depression scores.

**Conclusion:**

These findings underscore the critical role of depression as a mediator in the CP‐AD relationship, which is further exacerbated by adverse SDH in older adults from LA. Addressing social disparities and improving access to mental health and pain management services are essential for mitigating dementia risk and improving overall health outcomes in this vulnerable population.

